# Validated predictive equations based on tibial length in estimating height for children with cerebral palsy for 2–18 years, across all GMFCS levels

**DOI:** 10.1017/jns.2021.101

**Published:** 2021-12-17

**Authors:** Mahnon Suria Mokhy, Rosita Jamaluddin, Abd Rasyid Ismail, Norhasmah Sulaiman, Siti Nur ‘Asyura Adznam, Intan Hakimah Ismail, Malina Osman

**Affiliations:** 1 Department of Dietetics, Faculty of Medicine and Health Sciences, Universiti Putra Malaysia, Serdang, Selangor, Malaysia; 2 Department of Dietetics, Hospital Melaka, Malaysia; 3 Department of Nutrition, Faculty of Medicine and Health Sciences, Universiti Putra Malaysia, Serdang, Selangor, Malaysia; 4 Malaysian Research Institute on Ageing (MyAgeing), Universiti Putra Malaysia, Serdang, Selangor, Malaysia; 5 Department of Paediatric, Faculty of Medicine and Health Sciences, Universiti Putra Malaysia, Serdang, Selangor, Malaysia; 6 Department of Microbiology and Parasitology, Faculty of Medicine and Health Sciences, Universiti Putra Malaysia, Serdang, Selangor, Malaysia

**Keywords:** Actual height, Cerebral palsy, Estimated height, Predictive equation, Tibia length

## Abstract

Children with cerebral palsy (CP) typically suffer from congenital deformities, such as scoliosis and contractures, therefore, it is a challenge to measure the stature of CP children. Studies have suggested that predictive equations based on tibia length (TL) may be used as an alternative method in measuring the actual height or stature. The present study aimed to develop and validate predictive equations based on TL for CP children in Malaysia across all five levels of gross motor functions (GMFCS I to V) through a cross-sectional study. All subjects were recruited from Hospitals and Community-Based Rehabilitation (CBR) in the central and southern regions of Malaysia. Two predictive equation models were developed using multiple linear regression. For Model 1, the predictive equation was developed based on TL. On the other hand, Model 2 was developed based on TL with age was included. A flexible Seca measuring tape was used to measure the stature and TL. CP children aged 2–18 years were classified into the equation development group (EDG), *n* 177 and the validation group (VG), *n* 139. Model 1, Height = 32⋅3 + 3⋅14 (TL), demonstrated a strong correlation with the actual height (*R*^2^ 0⋅834), small SEE (1⋅42), and high intra correlation coefficient (0⋅929). The findings suggested that Model 1 was more accurate in estimating the height of CP children aged 2–18 years. This model was shown to suit the Malaysian population and applicable across all GMFCS levels.

## Introduction

Assessing the nutritional status among cerebral palsy (CP) children is challenging due to the difficulty in obtaining reliable measurements, such as weight and height. Several factors contribute to this difficulty, such as scoliosis, muscle weakness, spasticity, contractures, bone deformities, uncooperative individuals and other practical problems^(1)^. This shortcoming may result in inaccurate measurements, leading to misinterpretation of the nutritional status^(2)^. It is important to note that CP children have five levels of gross motor functions (GMFCS I to V)^(3)^. The higher the level of GMFCS (i.e. level IV and level V), the harder it is to measure the height of the CP children^(4)^.

One way to overcome this challenge is by using the segmental length equations that have been developed as an alternative method to measure the actual height or stature. Several predictive equations have been developed to estimate the height of CP children by measuring their segmental length^(5,6)^. One of the equations is the knee height equation. This equation, however, is not practicable to measure the height of CP children with severe knee and ankle joint contractures^(7)^.

Another way to determine segmental length is using a predictive equation based on tibia length (TL). This equation is more practical to estimate the height of CP children with lower limb joint contractures or scoliosis^(6)^. Additionally, it is more feasible to measure TL as compared to other segmental lengths (e.g. knee height, ulna length, arm span and demi span). Furthermore, previous studies showed a strong positive correlation between TL and estimated height *R*^2^ 0⋅81^(6)^ and *R*^2^ 0⋅94^(5)^.

However, the limitations with previous studies that used predictive equations based on TL indicated that the equations were age-specific and not applicable for all GMFCS levels. For instance, Kihara *et al.*^(6)^ used TL measurement to estimate the height of Japanese children with moderate to severe CP aged 3–12 years only. Additionally, Stevenson^(5)^ used TL measurement to estimate the height of children with moderate CP aged up to 12 years old. In another study, TL measurement was used to estimate the height of children aged 3–18 years in China but only among healthy children^(8)^. In addition, these equations have not been validated as suitable for Malaysian CP children, and thus, the malnutrition may be over or underestimated.

The nutritional status of individuals with CP aged 2–20 years was assessed by referring to growth charts developed based on the GMFCS levels^(3)^. One of the indicators used in these growth charts is height measurement^(3)^. Therefore, it is crucial to accurately estimate the height of CP children when neither height nor weight can be measured^(9)^. The current predictive equation to estimate the height based on TL is only for CP children aged up to 12 years. To date, there has not been any predictive equation using TL that can be used to estimate the height of CP children and adolescents age up to 18 years and across all GMFCS levels.

Therefore, the first objective of the present study is to develop a predictive equation based on TL that is (i) suitable for CP children age 2–18 years and (ii) applicable across all levels of GMFCS. It is hoped that this equation can be applied to estimate actual height in the assessment of nutritional status using a CP growth chart (for age 2–20 years). Moreover, the TL equation developed in the previous studies may not be appropriate for the CP children in the Malaysian population. Thus, the second objective of the present study is to validate the predictive equation based on TL in the Malaysian population.

## Methods

### Participants

A total of 316 subjects were recruited in this cross-sectional study. The inclusion criteria for the present study were as follows: (i) age between 2 and 18 years, (ii) clinically diagnosed with CP and (iii) classified as GMFCS levels I to V. On the other hand, participants were excluded if they had any of the following criteria: (i) Down's syndrome, (ii) hydrocephalus, (iii) genetic disorder and (iv) chronic illness. Written informed consent was obtained from the parents of the participants before the recruitment process. Ethical approval for this study was obtained from the National Medical Research Registration (NMRR) and the Ministry of Health Malaysia (NMRR-17-3373-35721).

### Development and validation of predictive equations

The first phase of the study (Phase 1) involved developing predictive equations based on TL. Six hospitals in the central and southern regions of Malaysia were selected based on the following criteria: (i) state-level hospital, (ii) rehabilitation hospital and (iii) hospital with CP clinic. From these six hospitals, a total of 177 participants were randomly recruited from outpatient paediatric clinics. All participants in Phase 1 were assigned to the equation development group (EDG). Two predictive equation models were also developed in this phase.

In the second phase of the study (Phase 2), the two developed predictive equation models were validated using a cross-validation technique. A total of 139 CP children were recruited to validate the equations. These children were selected from nineteen community-based rehabilitation (CBR) centres located in the central and southern regions of Malaysia. All participants in Phase 2 were assigned to the validating group (VG).

### Anthropometric measurements

In the present study, anthropometric measurements were performed by two trained dietitians. One dietitian (evaluator) measured the subject, while another dietitian (recorder) recorded the measurements. Every measurement was taken to the nearest 0⋅01 cm and read aloud to the recorder. An evaluator measured each subject three times for each group, and the mean value was calculated for analysis.

### Height measurement and recumbent length

A standard stadiometer was used to measure the height of the subjects who were able to stand. On the other hand, a flexible Seca tape was used to measure the recumbent length of the subjects who were unable to stand. The recumbent length was taken by measuring the four segmental lengths: (i) from the top of the head to the acromion process of the shoulder, (ii) from the acromion process of the shoulder to the greater trochanter of the hip, (iii) from the greater trochanter of the hip to the lateral joint of the knee and (iv) from the knee joint line to the bottom of the heel^(6)^.

### TL measurement

TL was measured using a Seca flexible measuring tape. The measurement was taken from the medial tibia condyle superior border to the medial malleolus inferior border. The subjects’ knee and ankle were positioned at a 90-degree angle during the measurement^(5)^.

### Statistical analysis

The data were analysed using the Statistical Package for Social Sciences (SPSS) software version 25.0. Data normality was assessed using the Kolmogorov–Smirnov test, while demographic data were analysed using descriptive analysis and simple frequency. Pearson correlation analysis was used to assess the relationship between the outcome variables. The predictive equations for estimating height was developed using a linear regression model. The coefficient of determination (*R*^2^) value and standard error of the estimate (SEE) were analysed to determine the predictive ability of the equations. The intraclass correlation coefficient (ICC) (95 % CI) was used to concord between the actual height and the estimated height. The significant level was set at *P* < 0⋅05 for all the statistical tests.

## Results

### Demographic characteristics

The demographic data of the 316 subjects are tabulated in Table 1. The samples comprised of more male (56⋅9 %) than female (43⋅1 %). However, gender was almost equally distributed between the EDG and VG groups. A majority of the subjects were Malays (77⋅2 %), followed by Chinese (14⋅9 %), and Indians (7⋅9 %). Additionally, most of the subjects were in the GMFCS level V category (51⋅6 %).

**Table 1.** Demographic data showing gender, race and GMFCS level of the participants

Variables	All Subjects (EDG and VG) (*n* 316) *n* (%)	EDG (*n* 177) *n* (%)	VG (*n* 139) *n* (%)
Gender			
Male	179 (56⋅6)	99 (55⋅9)	80 (57⋅6)
Female	137 (43⋅4)	78 (44⋅1)	59 (42⋅4)
Race			
Malay	244 (77⋅2)	114 (64⋅4)	130 (93⋅5)
Chinese	47 (14⋅9)	39 (22⋅0)	8 (5⋅8)
Indian	25 (7⋅9)	24 (13⋅6)	1 (0⋅7)
GMFCS level			
Level I	57 (18⋅0)	42 (23⋅7)	15 (10⋅8)
Level II	48 (15⋅2)	25 (14⋅1)	23 (16⋅5)
Level III	24 (7⋅6)	10 (5⋅6)	14 (10⋅1)
Level IV	24 (7⋅6)	10 (5⋅6)	14 (10⋅1)
Level V	163 (51⋅6)	90 (50⋅8)	73 (52⋅5)

EDG, equation developing group; GMFCS, gross motor function; VG, validating group.

### Age and anthropometry measurements

The mean age of all subjects was 8⋅00 (4⋅13) years. As shown in Table 2, there was no significant difference in age and actual height between the EDG and VG groups. However, there were significant differences in TL between both groups (*P* = 0⋅009).

**Table 2.** Age, height and TL of the participants

Variables	All Subjects (*n* 316) Mean (sd)	EDG (*n* 177) Mean (sd)	VG (*n* 139) Mean (sd)	*t* statistic	*P*-value
Age in years	8⋅8 (4⋅19)	8⋅9 (4⋅08)	8⋅7 (4⋅35)	0⋅565	0⋅570
Actual Height	115⋅6 (19⋅83)	116⋅8 (20⋅39)	114⋅2 (19⋅08)	1⋅141	0⋅250
TL	26⋅2 (5⋅74)	26⋅9 (5⋅93)	25⋅3 (5⋅36)	2⋅625	0⋅009*

EDG, equation developing group; TL, tibia length; VG, validating group.

* Significant at *P* < 0⋅05.

### Correlation between age, height and TL of the EDG

Pearson product-moment correlation coefficient (*r*) was used to determine the relationship between TL, age and actual height. There was a significant positive correlation between actual height and TL (*r*  0⋅91, *P* < 0⋅001). Additionally, age showed a strong positive correlation with actual height (*r* 0⋅83, *P* < 0⋅001; Table 3) and TL (*r* 0⋅81, *P* < 0⋅001; Table 3).

**Table 3.** The correlation between TL, age and actual height

Relationship	*r*	*P*-value
TL and actual height	0⋅91	<0⋅001
Age and actual height	0⋅83	<0⋅001
Age and TL	0⋅81	<0⋅001

TL, tibia length.

### Development of predictive equations based on TL

Model 1 shows the predictive equation for estimating height based on TL using the regression model. The linear regression equation for Model 1 is shown as follows:
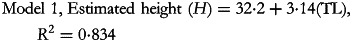


The age factor was later added to the above equation to improve the accuracy of the predictive equation in Model 2 using multiple linear regression. The linear regression equation for Model 2 is shown in the following equation:
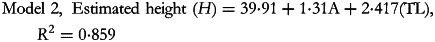
The prediction equations for Model 1 and Model 2, with a 95 % reference range, are shown in Table 4. According to Guilford's rules of thumb, the coefficient value between 0⋅7 and 0⋅9 indicates a strong relationship between two variables. Our results showed that the predictive power of each equation was strongly correlated with the TL of Model 1 (*R*^2^  0⋅834) and Model 2 (*R*^2^ 0⋅859).

**Table 4.** Mean estimated height, *R*^2^ and SEE of the VG group calculated using Model 1 and Model 2 equations

Parameter (*n* 139)	*R*^2^	SEE	Estimated height (*n* 139)			
					95 % CI of mean	
			Mean	sd	Lower	Upper
Actual Height			114	19⋅08		
Model 1: TL	0⋅834	1⋅42	112	16⋅81	109	114
Model 2: TL and A	0⋅859	1⋅44	94*	17⋅05	91	97

A, age; SEE, standard error of the estimate; TL, tibia length.

### Actual height and estimated height

**Fig. 1.**
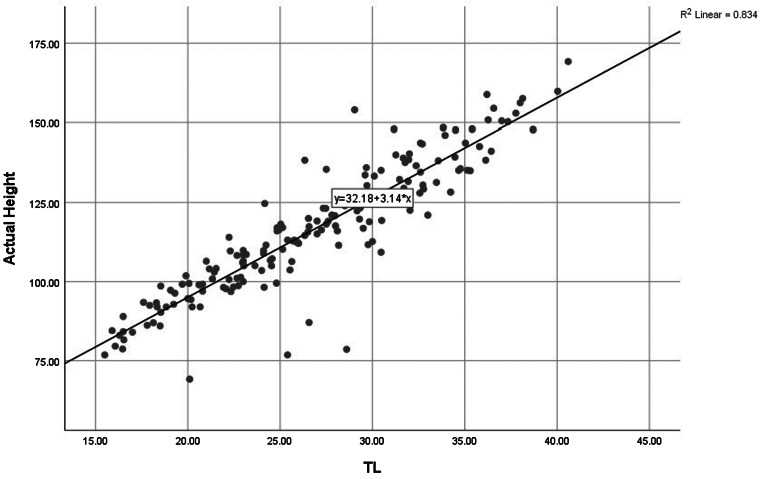
The regression equation describing the relationship between actual height and tibial length for CP children aged 2–18 years.

For validation purposes, the estimated height of the VG group was calculated using the equations of Model 1 and Model 2. The results are tabulated in Table 4. The scatter diagram (Fig. 1) illustrates that the correlation between TL measurements and standing height is calculated using the Model 1 equation.

### Validating the estimated height based on TL

The mean difference between the actual height and the estimated height for Model 1 was 2⋅60. On the other hand, the mean difference between the actual height and the estimated height for Model 2 was 20⋅23, which was larger than the Model 1. The results of Pearson's correlation analysis and ICC in Table 5 indicated a relationship between the actual height and TL equation (Model 1 and Model 2). The degree of agreement was computed using the ICC to demonstrate the relationship between the actual height and TL measurements. The results of this analysis are tabulated in Table 5. The ICC for Model 1 was 0⋅929, while the ICC for Model 2 was 0⋅560. Additionally, the correlation between TL and the estimated height was higher in Model 1 (*r* 0⋅94) as compared to Model 2 (*r* 0⋅92).

**Table 5.** Power of validation of the equations

Equation model	Mean difference	se	95 % CI of mean difference		*r*	ICC	95 % CI of ICC	
			Lower	Upper			Lower	Upper
Model 1	2⋅60	0⋅570	1⋅460	3⋅740	0⋅94	0⋅929	0⋅902	0⋅949
Model 2	20⋅23	0⋅627	18⋅99	21⋅47	0⋅92	0⋅560	-0⋅053	0⋅855

ICC, intraclass correlation coefficient; *r*, Pearson's correlation coefficient.

## Discussion

Measuring height or stature accurately in CP children is still difficult, but it is even more difficult in severe CP children. TL is one of segmental length that has been used in the development of predictive equations^(10)^. The study found that predictive equation based on TL has good accuracy in estimating the height of children with CP, regardless of the deformities^(6)^. Other studies also found that the TL measurement was reliable and strongly correlated with actual height^(11,12)^. In relation to these studies, we have developed two predictive equations models based on TL that can be used among CP in the Malaysian population. In Model 1, the equation was developed based only on TL. On the other hand, Model 2 was developed based on TL and age. Our study found a strong correlation (*r* 0⋅94, *P* < 0⋅001) between TL and the actual height.

It was found that the value of *R*^2^ was lower in a study that included age as an additional covariate in the predictive equation than in the studies that did not^(5,6)^. In our study, we found that when age was included as a covariate, the correlation strength between actual height and TL (*R*^2^ 0⋅859) was slightly higher but not significantly different than when age was not included (*R*^2^ 0⋅834). Our finding supports another study that included age as a covariate to the predictive equation based on TL which also found that age did not significantly affect the estimated height^(13)^. However, it can be said that both models had strong relationships with TL.

The present study also showed that the mean actual height of all subjects was 114 cm. The mean of estimated heights using both prediction equations in Model 1 and 2 was less than mean actual height. In model 1, the mean estimated height was 112 cm, which is closer to mean actual height. The difference might be due to involuntary movement and child position during the measurement which resulting approximately ±2 cm differences^(14)^. Regardless of the presence of joint contracture or scoliosis, measuring the tibia length with a measuring tape was simple, resulting in a small difference between the estimated and actual height in Model 1. This finding is similar with previous studies, where tibia length can be used to estimate height among CP children^(5,6,15)^.

On the other hand, the present study found that the mean estimated height calculated using the Model 2 was 94 cm which has 17⋅5 percent difference with actual height, when age was added. The difference could be due to age bone delay among CP children^(14)^. Because of the large mean difference in Model 2, the height measurement may be underestimated. These findings indicated that adding age as an additional covariate to the predictive equation produced a large mean difference in the estimated height. This explanation justified the low mean value of the estimated height in Model 2. According to one study, it may be useful to have methods for predicting height that take into account the height loss which was observed in CP children who are unable to stand^(10)^.

Apart from that, the result also showed that the SEE of Model 2 was greater than that of Model 1. This result indicated that the Model 2 equation produced a greater error when estimating height. A possible explanation for this finding was that congenital deformities were more severe in older CP children^(16)^. In support of this explanation, a study showed that 68 % of CP children had a bone age delay of more than a year^(14)^. In addition, another study found a large difference in the bone age of CP children, especially in those with severe oral motor dysfunction^(17)^.

Lastly, it is important to note that the TL measurements can vary with ethnicity and race^(13,18)^. Therefore, it is crucial to consider these two factors when generalising the findings to the population. Furthermore, previous studies in different populations reported inconsistent findings on the correlation between standing height and TL. For instance, a study among caucasian CP children reported a very strong correlation between standing height and TL (*R*^2^ 0⋅94)^(5)^. Another study showed a strong correlation (*R*^2^ 0⋅81) between the standing height and TL among Japanese children with moderate to severe CP^(6)^. Additionally, a study among Indian children with disabilities showed a strong correlation (*R*^2^ 0⋅72) between the standing height and TL^(12)^. In the present study, the ICC analysis was performed to validate the regression equation of the two models. The results showed that the mean difference between the actual height and the estimated height was larger in Model 2 than Model 1. Additionally, the results showed that the ICC of Model 1 was higher (ICC = 0⋅929) than Model 2 (ICC = 0⋅56). This finding indicated that Model 1 has better agreement as compared to Model 2, which suggested that Model 1 is more accurate in estimating the height of CP children aged 2–18 years old.

The present study, however, has several limitations that should be discussed. Firstly, the sample size of the present study was small and involved only the subjects in the central and southern region of Peninsular Malaysia. Therefore, the findings of the present study could not be generalised to the whole population. Future studies may consider using a larger sample size. Secondly, race (i.e. Malay, Chinese and Indian) were not equally distributed between the EDG and VG groups. This shortcoming may potentially bias the TL measurements of the present study.

In conclusion, the novelty of the present study was in the development and validation of two predictive model equations for estimating the height of CP children from age 2 to 18 years. Additionally, these predictive model equations have been validated for the Malaysian population and are suitable across the GMFCS levels. Between the two models, Model 1 demonstrated a strong correlation with the actual height (*R*^2^ 0⋅834), small SEE (1⋅42) and high ICC (0⋅929). Therefore, we recommend the use of this model in estimating the height of CP children all across the GMFCS level in Malaysia. Finally, it is hoped that these equations may serve as alternative methods to estimate the height in nutritional and clinical assessments.
